# The effectiveness of knowledge-sharing techniques and approaches in research funded by the National Institute for Health and Care Research (NIHR): a systematic review

**DOI:** 10.1186/s12961-024-01127-5

**Published:** 2024-04-02

**Authors:** Helen Baxter, Lindsay Bearne, Tracey Stone, Clare Thomas, Rachel Denholm, Sabi Redwood, Sarah Purdy, Alyson Louise Huntley

**Affiliations:** 1 Evidence and Dissemination, National Institute for Health and Care Research, Twickenham, United Kingdom; 2 Population Health Research Institute, St George’s, University of London, London, United Kingdom; 3 National Institute for Health and Care Research, Applied Research Collaboration West (NIHR ARC WEST), University Hospitals Bristol and Weston NHS Foundation Trust, Bristol, United Kingdom; 4 National Institute for Health and Care Research, Health Protection Research Unit in Behaviour Science and Evaluation (NIHR HPRU BSE), University of Bristol, Bristol, United Kingdom; 5 National Institute for Health and Care Research, Bristol Biomedical Research Centre (NIHR BRC), University of Bristol, Bristol, United Kingdom; 6 Population Health Sciences, Bristol Medical School, University of Bristol, Bristol, United Kingdom; 7 Centre for Academic Primary Care, Bristol Medical School, University of Bristol, Bristol, United Kingdom

**Keywords:** Systematic review, Knowledge sharing, Mechanism, Knowledge creation, NIHR

## Abstract

**Background:**

The National Institute of Health and Care Research (NIHR), funds, enables and delivers world-leading health and social care research to improve people’s health and wellbeing. To achieve this aim, effective knowledge sharing (two-way knowledge sharing between researchers and stakeholders to create new knowledge and enable change in policy and practice) is needed. To date, it is not known which knowledge sharing techniques and approaches are used or how effective these are in creating new knowledge that can lead to changes in policy and practice in NIHR funded studies.

**Methods:**

In this restricted systematic review, electronic databases [MEDLINE, The Health Management Information Consortium (including the Department of Health’s Library and Information Services and King’s Fund Information and Library Services)] were searched for published NIHR funded studies that described knowledge sharing between researchers and other stakeholders. One researcher performed title and abstract, full paper screening and quality assessment (Critical Appraisal Skills Programme qualitative checklist) with a 20% sample independently screened by a second reviewer. A narrative synthesis was adopted.

**Results:**

In total 9897 records were identified. After screening, 17 studies were included. Five explicit forms of knowledge sharing studies were identified: embedded models, knowledge brokering, stakeholder engagement and involvement of non-researchers in the research or service design process and organisational collaborative partnerships between universities and healthcare organisations. Collectively, the techniques and approaches included five types of stakeholders and worked with them at all stages of the research cycle, except the stage of formation of the research design and preparation of funding application. Seven studies (using four of the approaches) gave examples of new knowledge creation, but only one study (using an embedded model approach) gave an example of a resulting change in practice. The use of a theory, model or framework to explain the knowledge sharing process was identified in six studies.

**Conclusions:**

Five knowledge sharing techniques and approaches were reported in the included NIHR funded studies, and seven studies identified the creation of new knowledge. However, there was little investigation of the effectiveness of these approaches in influencing change in practice or policy.

**Supplementary Information:**

The online version contains supplementary material available at 10.1186/s12961-024-01127-5.

## Background

Academic research has little influence on the commissioning, design and delivery of health care services [1–3]. Stakeholders, including patients, are currently not consulted sufficiently for research to be genuinely informed by their experiences [4, 5]. This is of concern to research funders globally, who have a remit to fund health and social care research that improves people’s health and wellbeing [6]. Knowledge mobilisation is a generic term that refers to making knowledge ready for action and includes activities ranging from dissemination to co-production [7]. Other similar terms are often used such as knowledge translation, knowledge exchange and integrated knowledge translation (IKT). For the purposes of this review, the key element of knowledge sharing was focused on within the field of knowledge mobilisation to explore knowledge mobilisation as an intervention and an active process, within research studies. Exploration of the lack of integration between researchers and stakeholders within the fields of knowledge mobilisation and implementation has highlighted that knowledge sharing needs to be a two-way process and not, as previously accepted, a linear one [8–11]. This shift in understanding has been driven through a recognition of the complexity and messiness inherent in bringing together different communities to develop a common or shared understanding [3, 12]. Consequently, activities to improve knowledge sharing and implementation have shifted away from targeting research findings towards patients, practitioners and policy makers and been replaced with techniques to encourage two-way knowledge sharing and co-production [9, 13–15]. A variety of theories, models and frameworks have been used to support this two-way process, with varying degrees of success [16, 17].

Knowledge mobilisation is defined by the NIHR as ‘sharing knowledge between different communities to create new knowledge to catalyse change’ [18]. There is consensus that if knowledge is shared between two or more communities, it can result in the creation of new knowledge, which has a greater likelihood of leading to change within practice or research [7, 19–21]. Change that can be linked back to original research findings or outcomes is often referred to as research impact [22–24]. Techniques and approaches that have been developed to follow this mechanism of knowledge sharing include, models of embedded researchers or practitioners, use of knowledge brokers, stakeholder engagement, organisational collaborative partnerships and the involvement of stakeholders in the research or service design process itself. For example, embedded models can facilitate the knowledge sharing process by a researcher or health care practitioner leaving their home organisation to work in a host organisation, thereby increasing the opportunities for sharing knowledge between the two organisations. The underlying premise is that it is through people and their interactions that knowledge is shared and by increasing the proximity of individuals this can facilitate interactional opportunity [10, 25, 26]. They may be hosted by one organisation, but their function is to work between the organisations to facilitate knowledge sharing [27–29]. Stakeholder engagement, when conducted for two-way knowledge sharing, involves inviting stakeholders to share knowledge at specific meetings, workshops and events [30]. Involving stakeholders in the research or service design process as equal decision makers, advisers and informed representatives of their community, can also follow two-way knowledge sharing [21, 31, 32]. An additional mechanism is knowledge sharing at an organisational level, where collaborative partnerships are formed [33].

In the United Kingdom, the National Institute of Health and Care Research (NIHR) awards around £1 billion in research funding per year and, along with other funders, has a strong remit to reduce the research to practice and policy gap [34]. Yet, to date, there has been limited research that systematically explores and identifies the knowledge sharing techniques and approaches in the NIHR portfolio of research studies. One review examined the mechanisms and pathways to impact of NIHR funded public health research (Boulding, Kamenetzky et al. 2020). It explored the mechanisms and pathways reported on Research fish (a database for researchers to document impact related activities) and triangulated this with qualitative data exploring the researchers’ perspectives of the impact of their research. The authors concluded that the standardised measures were not capturing impact in localised settings or longer-term impact [23]. A second study explored the public health researchers’ perspectives on impact reporting and highlighted a need for funders to identify their expectations of the impact resulting from the research they fund and to increase their support for knowledge mobilisation activities [24]. These studies highlighted the need for researchers to have a clearer understanding of the knowledge mobilisation techniques and approaches to inform pathways to impact and focused on NIHR health funding streams [23, 24]. To our knowledge, there has been no systematic review that describes the knowledge sharing techniques and approaches that have been applied in NIHR funded research nor synthesises their effectiveness.

This review aimed to answer the following questions: (1) Which knowledge sharing techniques and approaches have been included in NIHR funded health research? (2) How effective are these knowledge sharing techniques and approaches in creating new knowledge that can lead to changes in practice and research?

## Methods

The protocol for this systematic review was registered on the International Prospective Register of Systematic Reviews (PROSPERO, CRD42020171293; reported in accordance with the Preferred Reporting Items for Systematic Reviews and Meta-Analyses [35]). A restricted systematic methodology was chosen to balance methodological rigour with the resources available [36].

### Search strategy

Electronic databases MEDLINE via OVID and The Health Management Information Consortium, which is a compilation of data from two sources, the Department of Health’s Library and Information Services and King’s Fund Information and Library Services, were searched from inception to 24.4.20 for published studies, which was then updated and rerun on the 1.7.22. The search strategy was based on the terms for the intervention (knowledge sharing techniques and mechanisms, including terms for knowledge transfer, exchange and translation) and population (researchers with patients, clinicians or health services managers) (Additional file 1: Search Strategy). Additional references were identified from reference lists of included full papers.

### Eligibility criteria

This systematic review included studies that described knowledge sharing between researchers with patients, members of the public, clinicians, health service managers (i.e. commissioners, policy makers and hospital managers) or voluntary agencies, that were funded by the NIHR (Table 1). Knowledge sharing was defined as ‘any interactional activity through any medium (including in person, email, telephone, etc.) that involves knowledge sharing about healthcare’. For the purposes of this review, knowledge sharing techniques and mechanisms were considered as an intervention, i.e. ‘the act or an instance of intervening’ [37], where an explicit knowledge-sharing approach had been adopted in contrast to the established process of knowledge remaining within one community. The setting was defined as any healthcare setting, e.g. primary, secondary, tertiary health care services and public health. The outcome was defined as the use of evidence in policy and practice or the involvement of stakeholders in the research process. Where relevant, studies were included irrespective of comparator group. All study designs were included, except protocols and reviews of literature. Only studies published in the English language were included. Studies were excluded if they did not describe knowledge sharing between researchers and a stakeholder group, e.g. describing knowledge sharing between two other stakeholder groups (e.g. clinicians with health service managers, clinicians with patients and patients with health service managers).

**Table 1:** Inclusion and exclusion criteria

Inclusion criteria	Definition	Exclusion criteria
Population	Researchers with clinicians or health service managers (definition of commissioners, policy makers and hospital managers) or patients/public contributors, including community leaders	Stakeholder to stakeholder
Intervention	Any shared activity through any medium (email or telephone) that involves knowledge sharing (or transfer or mobilisation) about healthcare; looking for evidence of a two-way interaction	Co-research, as participating in research process but not knowledge sharing
Control	Any control group if present	
Outcome of interest	Primary – relevant techniques or approaches to inform the practice of knowledge sharing Secondary – have been deemed successful or not	
Design	To explore how a technique or approach is working. Either detailed description or an additional methodology that explores the processes of the technique or approach	

### Study selection

Records were exported and deduplicated in Endnote and then imported to Covidence for screening [38, 39]. The title and abstract screening was conducted by one reviewer (H.B.), with a 20% sample independently screened by one of two reviewers (C.T. and R.D.). Any discrepancies were resolved by discussion. A third reviewer (A.H.) arbitrated if needed. Full text screening was conducted by one reviewer (H.B.) with a 20% sample independently screened by one of two reviewers (T.S. and L.B.); any discrepancies were resolved by discussion. A third reviewer (S.R.) arbitrated if needed.

### Data extraction

Data from included studies were abstracted by one reviewer (T.S.) into a data extraction form, which was piloted a priori on 10% of the included studies (S.P.) and checked for accuracy by a second reviewer (H.B.). Extraction included: study design, author name, author, year, aims, population, intervention/approach and a detailed intervention description. In some instances, studies contained a knowledge sharing element, which was not the primary focus or outcome of the study. In these cases, the detailed description of this element of the study was extracted as the technique or approach. A modified template of the TiDieR checklist was used [40]. The data were extracted on the design, presence of an evaluation, use of theory or goal, procedures, materials used, context influencing factors, tailoring modifications and assessment of outcome and applicability.

### Quality appraisal

Quality appraisal was conducted independently by T.S. with a 20% sample of included studies, which were reviewed by H.B., followed by discussion for any discrepancies. The Critical Appraisal Skills Programme (CASP) qualitative checklist, Critical Appraisal Skills Programme (2018) [41] was used where appropriate. The CASP qualitative checklist includes two screening question (yes/no) and an additional eight questions (yes/ no/can not tell) if the response to both screening questions were ‘yes’. As outlined by Long and French, the quality of studies was assessed with a focus on the rigour of the data analysis, with consideration of the trustworthiness of the results given [41]. Using this focus with the overall score from the checklist, the studies were categorised to be of high, moderate or of lower quality.

### Data synthesis

A narrative synthesis method was adopted, as it includes a formal analytical process of synthesis to generate new insights [42]. This narrative synthesis focussed on four key elements: (1) identification of a theory of change. In this review, knowledge sharing as a mechanism to facilitate change was used to explain the anticipated process. (2) Development of a preliminary synthesis of the findings of included studies. A preliminary synthesis was conducted to organise the results of the included studies and identify any factors that influenced the results reported. This was conducted by developing initial descriptions of the results of the included studies, which were then organised to describe patterns, so that the factors impacting on the mechanisms of the intervention could be identified. (3) Exploring relationships in the data. The studies were explored for relationships within and between studies, which involved a process of concept mapping supported by qualitative case descriptions. In particular, the studies were examined for instances where similar mechanisms may be at work even though the overall approach may be described differently. This process was initiated by H.B. in categorising the data under overarching themes based on the mechanism of knowledge sharing, which were refined further through discussion and reflection with L.B. and T.S. into subheadings. (4) Assessing the robustness of the synthesis. An assessment of the robustness of the synthesis was made and only studies that reached a minimum standard of methodological quality assessed by T.S. were included in the final synthesis [43].

## Results

### Study selection

**Fig. 1:**
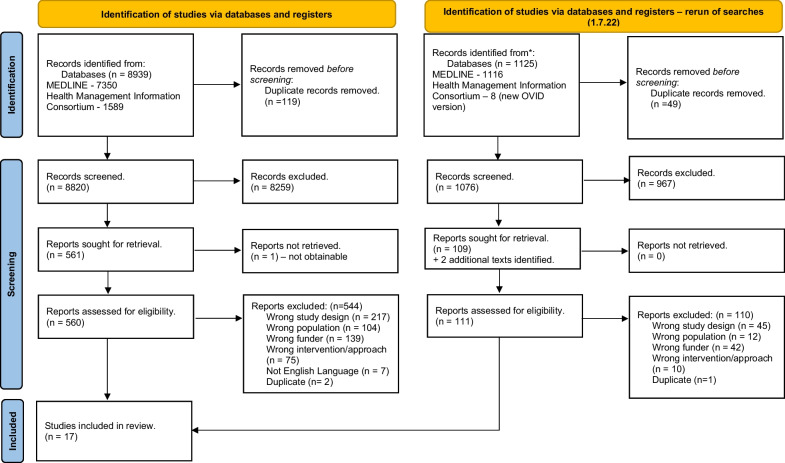
PRISMA diagram

In total, 9897 records were identified after deduplication. A total of 697 full-text studies were screened and 17 studies were included [20, 44–59] (Fig. 1).

### Study characteristics

The characteristics of the included studies are shown in Table 2. These were the author, year, aims, population, knowledge sharing technique or approach, mechanism of knowledge sharing and outcome (new knowledge or change in practice or research).

**Table 2:** Characteristics of included studies

Author, year	Aims	Population	Intervention/two-way knowledge sharing technique or approach	Mechanism of knowledge sharing	Outcome (new knowledge or change in practice or research)
Batchelor 2013	To identify and prioritise eczema treatment uncertainties that are of importance to patients who have the disease, their carers and the health care professionals who treat them	40 Researchers, patients, carers and clinicians	*Stakeholder engagement* Priority Setting Partnership as part of a James Lind Alliance. The authors used a modified version of the James Lind Alliance approach, by including researchers as participants in the workshop phase of the approach. The approach was also extended by including the discussion of research questions and not just the generation of prioritized treatment uncertainties	Workshop taking place over one day, where participants went into four independently facilitated groups, which were equally balance across the population (e.g. patients and clinicians)	Discussion of six prioritised treatment uncertainties leading to 13 potential research questions* (new knowledge)* Evidence of change in research or practice was outside of scope of study
Clarke 2019	To assess how co-produced research is conditioned by the emergence of group unity and a shared sense of belonging	Four project teams and their wider stakeholders Researchers, patients, carers, clinicians, health service managers, local authority and representatives of the third sector	*Involvement within the research process* Involvement of stakeholders in the research process itself through meetings and other project related interactions	Routine encounters both formal and informal from the early stages of project design, start‐up through access negotiations, data collection, analysis and dissemination	Demonstrated how inclusivity is generated and maintained through co-production Evidence of change in research or practice was outside of scope of study
Cooke 2015	To identify the lessons learned from one collaboration and leadership in Applied Health Research and Care in relation to on-going collaborative research priority setting	Detail not given Researchers, clinicians and health service managers	*Stakeholder engagement* Collaborative priority setting (CPS), using three techniques of (1) trusted historical relationships, (2) platforms for negotiation and planning and (3) formal methods of consensus	Three techniques of knowledge sharing were used between researchers and stakeholders. Only the co-production workshops, categorised as (3) formal methods of consensus, led to new knowledge	Two projects were co-designed leading to joint grant capture* (new knowledge)* Evidence of change in research or practice was outside of scope of study
Devonport 2018 (not included in final synthesis)	To present a reflective account of patient and public involvement (PPI) in the development of obesity and binge eating research	16 Researchers, patients, clinicians and a member of the public	*Stakeholder engagement* Four Patient Advisory Group meetings	Insufficient detail on process of two-way knowledge sharing	Critical learning points identified on how to improve involvement of patients
Gerrish 2014	To evaluate the success of knowledge transfer capacity development secondments from the perspective of multiple stakeholders	Detail not given researchers, clinicians and healthcare managers	*Embedded models (practitioners)* Fourteen secondments of 6–24 months duration of nurses into knowledge transfer teams	Secondees worked alongside experienced team members who were leading knowledge transfer initiatives	New solutions were reported as a result of sharing clinical and academic knowledge* (new knowledge)* “..secondee brought ideas back to the workplace with audit results showing that nutritional referrals in the secondee’s clinical area had improved and were higher than those on comparable wards.” P.214* (evidence of change)*
Gillard 2012	To reflect on the extent to which knowledge was co-produced through qualitative analysis and to consider the implications of research co-production for study findings	17 Researchers, patients, carers, clinicians and health service managers	*Involvement within the research process* Patients and carers were involved in the data collection, analysis and interpretation of a qualitative study of mental health. Feedback conferences were also used	Patients and carers were involved throughout research process and given an equal voice in decision making within the research study	Discussion with patient and carers was reported as directly affecting research findings* (new knowledge)* Evidence of change in research or practice was outside of scope of study
Guell 2017	To explore how stakeholders assessed, negotiated and intended to apply multi-sectoral evidence in policy and practice at the intersection of transport and health	41 Researchers, local authority managers and representatives of the third sector	*Stakeholder engagement* An end of project stakeholder forum to present and discuss findings	Presentation of the study and key findings, followed by stands in a ‘marketplace’ format to facilitate discussion with members of the research team and other attendees. A plenary session to identify key learning implications for policy and practice	Knowledge identified in how to communicate across the different sectors but no new knowledge generated Evidence of change in research or practice was outside of scope of study
Hutten 2015	A priority-setting method for evidence-based service development to reconcile research with multiple stakeholder views	40 Researchers, service users, carers, clinicians, health service managers and commissioners	*Stakeholder engagement* Researchers and stakeholders participated in three workshops to review evidence and generate service improvement ideas	Two workshops to review the evidence from two research projects, which generated twenty suggestions for service improvements that were discussed and debated in a final consensus workshop	Knowledge was generated from a consensus for eight suggestions for implementation* (new knowledge)* Evidence of change in research or practice was outside of scope of study
Irving 2018 (not included in final synthesis)	To describe the process of involving patients and public representatives in identifying, prioritizing and refining a set of outcome measures that could be used to support ambulance service performance measurement	18 Researchers and members of the public	*Stakeholder engagement* An event was held that was organised with members of the public, as an engagement event for members of the public. A structured process of voting using technology was also used	Insufficient detail on process of two-way knowledge sharing	Event offered opportunities for more interactive engagement and personal contact with stakeholders. It also extended the influence of the public contributors in the study and build capacity for their involvement
Knowles 2021	To explore and evaluate the potential of a participatory codesign method as a mechanism of knowledge sharing	12 One researcher and eleven members of the public	*Involvement within the service design process* Public contributors were involved in a service design process that was facilitated and supported by a researcher	Ten participatory co-design workshops were held, using activities including narrative methods and modelling methods	Approach generated hybrid knowledge that reflected a merging of different ways of knowing and understanding* (new knowledge)* Evidence of change in research or practice was outside of scope of study
Redwood 2016	To describe and examine the development and establishment of micro-level operating units (health integration teams) of a locally evolved structural partnership of health organisations and academic institutions	Individuals from seven organisations, two universities, four provider organisations and one commissioning organisation	*Organisational collaborative partnership* Health integration teams formed in response to fragmentation within the commissioning of services and a lack of system leadership. Also, an initiative to promote evidence-based practice in commissioning and service delivery and a forum for integration	Process of change identified through four mechanisms (1) whole system engagement, (2) collaboration, (3) integration and (4) innovation	Knowledge was generated through the integration of the organisations in identifying solutions to challenges within the system* (new knowledge)* Evidence of change in research or practice was outside of scope of study
Shipman 2008	To identify major concerns of national and local importance in the provision, commissioning, research, and use of generalist end of life care	30 Researchers, clinicians, service commissioners, policy makers and user groups	*Stakeholder engagement* A national consultation and prioritising exercise using a modified form of the Nominal Group Technique	Five consultation meetings were held in each area for participants to discuss and clarify issues and prioritise research themes; non-attendees participated by telephone or email	Knowledge was shared, but it was reported that little consensus was reached. Several research questions were generated Evidence of change in research or practice was outside of scope of study
Smith 2015	To understand how researchers and health service managers made sense of new ways of working. To design and conduct a developmental evaluation of the collaborative aspects on vascular disease prevention in primary care	Actual number unclear Researchers, commissioning managers	*Organisational collaborative partnership* Collaboration between two universities and two healthcare organisations in a local area	Scheduled project management meetings were the observed to be the principal interface between partners from different organisations throughout the study	Boundary maintenance enabled the co-production of at some practical meaning or sense, but the generation of new knowledge was not described Evidence of change in research or practice was outside of scope of study
Van der Graaf 2019	To explore the challenges and opportunities to knowledge brokering in an institutional service	Five members of a knowledge brokering team and 150 researchers, public health teams, community sector workers and representatives of the third sector	*Knowledge brokering* Knowledge brokering within an established team at an organisational level	Conversations with policy and practice partners as part of the scoping of enquiries that the service received	Clear evidence of knowledge sharing process through this approach but new knowledge creation not described Evidence of change in research or practice was outside of scope of study
Vindrola-Padros 2019	To explore and analyse the ‘researcher-in-residence’ model of knowledge co-production	Three researchers in residence in three contexts, two NHS trusts and one commissioning organisation	*Embedded models (researchers)* A model of embedded researchers working inside healthcare organisations, operating as staff members, while also maintaining an affiliation with their academic institutions	As part of the local team, researchers negotiate the meaning and use of research-based knowledge to co-produce knowledge, which is sensitive to the local context	Clear evidence of knowledge sharing process through this approach but new knowledge creation not described Evidence of change in research or practice was outside of scope of study
Waterman 2015	To describe how knowledge transfer associates facilitated the implementation of evidence-based health care	Eight knowledge transfer associates who were researchers working across six project teams with clinicians and health service managers	*Knowledge brokering* Knowledge transfer associates worked across hospitals, primary care and community-based organisations to facilitate evidence-based health care	Facilitative role of the knowledge transfer associates created a knowledge sharing mechanism as they interacted with others	Evidence of knowledge sharing through approach but new knowledge creation not described Evidence of change in research or practice was outside of scope of study
Wright 2013	To describe how health practitioners were embedded as researchers within clinical practice and supported by a Collaboration for Leadership in Applied Health Research and Care	23 Seventeen allied health professionals working as researchers in clinical teams	*Embedded models (practitioners)* Practitioners were embedded within clinical teams and supported by academic mentors to increase research skills and build research capacity	Knowledge sharing occurred between practitioners and research mentors and also between practitioners in a researcher role with other members of clinical team	Practitioners used research knowledge gained to instigate changes in practice, but new knowledge was not evidenced as being created from knowledge sharing

### Quality appraisal

Five of the included studies were descriptive studies and could not be included in the quality appraisal process [20, 49, 51, 53, 57]. Of the remaining 10 studies, two were rated of moderate quality [47, 48] and eight were rated as high [44–46, 50, 52, 54–56]. Two studies could not be rated as they provided insufficient detail on the knowledge mobilisation intervention, so these were excluded from the final synthesis (Table 3).

**Table 3:** Quality appraisal of studies

Author, year	Formal evaluation	Clear statement of research aims?	Qualitative methodology appropriate?	Research design appropriate to address aims?	Theoretical under-pinning clear, consistent and coherent?	Recruitment strategy appropriate?	Data collected in a way that addressed the research issue?	Relationship with researcher considered?	Ethical issues considered?	Analysis methods/rigour?	Clear statement of findings?	How valuable is the research?
Clarke 2019	Yes	Yes	Yes	Yes	Yes	Yes	Yes	No	Yes	Yes	Yes	Yes
Cooke 2015	Yes	Yes	Yes	Yes	Yes	Yes	Yes	Yes	Yes	Yes	Yes	Yes
Gerrish 2014	Yes	Yes	Yes	Yes	Yes	Can not tell	Yes	No	Yes	Yes	Yes	Yes
Guell 2017	Yes	Yes	Yes	Yes	Yes	Yes	Yes	No	Yes	Yes	Yes	Yes
Knowles 2021	Yes	Yes	Yes	Yes	Yes	Can not tell	Yes	Yes	Yes	Yes	Yes	Yes
Redwood 2016	Yes	Yes	Yes	Yes	Yes	Yes	Yes	No	Yes	Yes	Yes	Yes
Smith 2015	Yes	Yes	Can not tell	Can not tell	Can not tell	Can not tell	Can not tell	No	Yes	Can not tell	Can not tell	Can not tell
Van der Graaf 2019	Yes	Can not tell	Can not tell	Can not tell	Yes	Can not tell	Yes	No	Can not tell	Yes	Yes	Can not tell
Waterman 2015	Yes	Yes	Yes	Yes	Yes	Yes	Yes	Yes	Yes	Yes	Yes	Yes
Wright 2013	Yes	Yes	Yes	Yes	Yes	Yes	Yes	Yes	Yes	Yes	Yes	Yes

### Types of knowledge sharing techniques and approaches

Five explicit forms of knowledge sharing studies were described in the included studies (Table 2). Three studies applied embedded models of researchers or practitioners [20, 44, 54], and two studies used knowledge brokering. [46, 47]. Stakeholder engagement approaches that applied two-way knowledge sharing were used in five studies. These were either priority setting consensus building workshops [51, 55, 57] or facilitated knowledge-sharing events [49, 52]. Three studies described approaches where non-researchers were involved in the research or service design process itself. One study did this with patients and members of the public in research projects and another with professionals [53, 56]. The approach of involving patient and public members was also used in another study to assist with service design [45]. Two studies examined organisational collaborative partnerships between universities and healthcare organisations [48, 50].

### Types of stakeholders

Of the stakeholder groups participating via these approaches, clinicians were involved in nine studies [44, 46, 49–51, 53–55, 57], and patients and the public were involved in six studies [45, 49, 51, 53, 56, 57]. Commissioners and policy makers were involved in six studies [20, 48–51, 56]. Four studies involved health care or service managers [51, 53, 54, 56]. Four studies also involved members of the voluntary sector [47, 49, 52, 56], and two studies included local authority staff [52, 56].

### Timing within research cycle

Six studies applied a knowledge-sharing approach to topic identification [44, 46, 47, 49, 50, 55], and one study extended topic identification to also defining the research question [57]. Five studies used a knowledge-sharing approach for the conduct of the research [20, 48, 53, 54, 56]. One study used knowledge sharing to facilitate the adoption of findings [52], and two studies used knowledge sharing for the production of service design [45, 51]. There were no studies that used a knowledge-sharing approach or technique for designing the research or preparing the funding application.

### Sources of NIHR funding

Eight of the studies were funded or supported by a Collaboration for Leadership in Applied Health Research (CLAHRC) [44, 46, 48, 50, 51, 54–56]. One study was funded by a Knowledge Mobilisation Research Fellowship [45], and one study reported support from both a Knowledge Mobilisation Research Fellowship and a CLAHRC [20]. Two studies were from the Health Services and Delivery Research funding stream [49, 53], one study was from multiple sources, including NIHR funding [47], one was funded by the Public Health Research Programme [52] and one was funded by Programme Grants for Applied Research Funding [57].

### Use of theory

Of the 15 studies, 6 studies drew upon or referred to a theory, theoretical basis or used a framework [20, 46–48, 50, 56], (Table 4). The theory most frequently drawn upon was that of Communities of Practice [60, 61], which was referred to by three of the studies to explain the process of knowledge sharing [20, 50, 56]. Two studies drew upon other theories to explain knowledge sharing as part of a co-production process. One referred to Ritual Theory [62] and the concept of Interaction Ritual Chain [56, 63], and the other used three theoretical lenses, the co-productionist idiom [64], interactionist currents within organisation studies [65, 66] and communication, argumentation and critique from a pragmatic perspective [67, 68], In Ref. [48]. Another study drew on the sociological theory of dramaturgical perspective [47, 69], and one study used the frameworks of why, whose, what and how [70] and PAHRIS [71] to explain their approach [46]. Only one study explicitly referred to a theory of change and outlined a potential process [50]. Nine studies did not use any theory or frameworks to explain or predict the knowledge sharing process leading to change [44, 45, 49, 51–55, 57].

**Table 4:** TIDieR intervention checklist

Author, year, country	Description of intervention	Rationale, theory or goal of intervention	Physical materials or informational materials used	Procedures/activities/processes used	Modes of delivery	Influencing factors and tailoring or modifications	Evaluation undertaken and assessment of outcome	Applicability, generalisability or external validity
Batchelor 2013	Workshop to review results of a prioritization exercise and to develop research questions based on prioritized uncertainties	Rationale – within a priority setting partnership, to use open engagement to discuss and to generate research questions by consensus	Summary information to provide contextual information about the topic	Workshop with different stakeholder groups Independently facilitated	Face-to-face Group Location unclear	Workshop had been modified from James Lind Alliance, Priority Setting Partnerships to include generation of research questions	No evaluation	Replicable across other groups and topic areas
Clarke 2019	Use of a co-production approach within research projects	Theory – ritual theory [1] and the Interaction Ritual Chain concept [2], to explain how inclusivity is established and maintained, as a key element of co-production	None reported	Project management group meetings at four project sites	Face-to-face Group Locations in three UK universities and local health and care providers	Projects selected on their ‘explicit use of co-production’	Ethnographic data were collected from observation, informal and semi-structured interviews Everyday rituals and routines were observed to generate and sustain inclusivity	Replicable across other groups and topic areas
Cooke 2015	Collaborative priority setting in a Collaboration for Leadership in Applied Health Research (CLAHRC)	Rationale – use of priority setting to build capacity and collaboration with stakeholders. Three strategies were described	Refreshments at meetings and workshops	a) Trusted historical relationships	a) Not described	None described	Qualitative semi-structured interviews, workshop, and documentary analysis Formal methods of consensus of co-production workshops were reported to have led to joint grant capture	Replicable as a whole approach across other organisations with resources similar to CLAHRCs
				b) Platforms for negotiation and planning	b) Special interest, steering and advisory groups			
				c) Formal methods of consensus	c) Delphi and Nominal Group Technique. Co-production workshops			
Gerrish 2014	Academic and clinical nurses were seconded into knowledge translation teams within a Collaboration for Leadership in Applied Health Research (CLAHRC)	Rational – to enhance knowledge translation (KT) expertise in KT teams and to provide capacity development opportunities to benefit CLAHRC partners	None reported	Not reported	Face-to-face, individually and in groups	None described	Pluralistic evaluation Focus groups, discussion groups and semi-structured interviews in two phases Secondees reported to have facilitated change in practice	Replicable in organisations with existing knowledge translation/mobilisation teams
Gillard 2012	Involvement of service users and carers in qualitative data analysis	Goal – to reflect on the extent to which knowledge was co-produced	Research data from semi structured qualitative interviews	Preliminary analysis, development and application of analytical framework, stakeholder conferences, asking questions of the qualitative data, writing up	Face-to-face in groups	None described	No evaluation	Replicable across other groups and topic areas
Guell 2017,	Stakeholder forum held on one occasion	Goal – to discuss relevant research evidence and observe knowledge exchange	Market stalls set up with over 20 publications to engage with	Market place format followed by a formal plenary session	Face-to-face, individually and in groups	None described	Ethnographic observation and semi-structured interviews Generated knowledge on how to communicate	Replicable across other groups and topic areas
Hutten 2015	Consensus workshops with range of stakeholders to identify and prioritise service improvement ideas	Goal – to demonstrate a method of generating and agreeing on service improvement priorities	Detailed briefing pack sent before the event Electronic voting technology	Short presentations, a question-and-answer session and process of voting on own individual priorities	Face-to-face in a group	None described	No evaluation	Replicable across other groups and topic areas
Knowles 2021	Participatory co-design workshops with patients and service users for service design	Rationale – that if authentic involvement was achieved this would lead to knowledge sharing	None reported but activities described suggest drawing materials	Ten co-design participatory workshops	Face-to-face in a group	None described	Collective in-action analysis, survey, focus group and field notes Learning generated on co-design process	Replicable across other groups and topic areas
Redwood 2016	Collaborative partnership between National Health Service partners, the city council and two universities	Theory – communities of practice theory (3) and a theory of change model developed to explain intervention	None reported	Collaborative stakeholder meetings for each micro-level team (Health Integration Team)	Face-to-face in groups	Influencing factors on organisational collaborative partnerships as a mechanism of knowledge sharing outlined through a theory of change	Document analysis and stakeholder semi-structured interviews	Difficult to replicate in areas without similar infrastructure and partnerships
Shipman 2008	Consultation meetings to clarify and prioritise research themes	Goal – to identify major concerns of national and local importance in the provision, commissioning, research and use of generalist end of life care	None reported	Consultation meetings held as part of a Nominal Group Technique, for participants to discuss and clarify and prioritise research themes,	Face-to-face in groups	Method of Nominal Group Technique was modified to generate ideas before the meeting and to allow those unable to attend to participate via email or telephone	No evaluation	Replicable across other groups and topic areas
Smith 2015	Organisational collaborative partnership between universities and health care organisations within a health care system	Theory – three theoretical lenses were used to explain the partnership working, the co-productionist idiom [4], interactionist currents within organisation studies [5, 6] and communication, argumentation and critique from a pragmatic perspective [7, 8], [9]	Formal project documents (boundary objects)	Project management group meetings and the use/negotiation around documentation	Face-to-face in groups	Study revealed the involvement of other organisations outside of the formal partnership	Observation, document analysis and postal questionnaire Identified how collaboration was being maintained by maintenance of boundaries rather than ‘blurring’ of them	Difficult to replicate in areas without similar infrastructure and partnerships
Van der Graaf 2019, United Kingdom	Knowledge brokering service between academics and health practitioners	Theory – use of ‘dramaturgical lens’ and ‘front and backstage’ in partnerships to explain knowledge brokering process [10]	None reported	Knowledge broker interactions with research requests from 150 + health, or social care sector representatives	Face-to-face, email and one-to-one conversations	None described	Auto-ethnographic evaluation of conversations from summary notes and emails Identified challenges and how these could be overcome by similar services	Difficult to replicate in areas without similar infrastructure and partnerships
Vindrola-Padros 2019, United Kingdom	The ‘researcher-in residence’ embedded model,	Rational – researchers in residence will negotiate the meaning and use of research and co-produce local context sensitive knowledge	None reported	Three aspects: (1) building relationships, (2) defining and adapting the scope of the projects and (3) maintaining academic professional identity	Face-to-face, individually and in groups	None described	No evaluation	Three case studies given, which aids replicability across other groups and topic areas
Waterman 2015, United Kingdom	Knowledge transfer associates, responsible for the facilitation of the implementation of evidence-based health care	Theory/framework—PARIHS model emphasising the facilitative function, and the use of a knowledge brokering framework [11, 12]	None reported	Knowledge transfer associates as part of a team responsible for implementing evidence-based health care	Face-to-face in groups	Knowledge transfer associate with a different theoretical underpinning perspective to a knowledge broker	Analysis of co-operative enquiry meetings and reflective diaries Identified factors that could support similar initiatives	Some potential to replicate model in organisations using evidence-based health care projects or equivalent
Wright 2013, United Kingdom	Referred to as knowledge brokers but described as embedded researchers within clinical teams (with a clinical professional backgrounds)	Rationale – that these allied health professionals would bridge the gap identified between research and practice through boundary spanning roles	None reported	Literature searches/reviews, empirical data collection and implementation of projects or processes with evaluation of outcome	Face-to-face, individually and in groups	None described	In-depth interviews, report and reflective diaries Identified increase in research skills in individuals, piloting of research findings in practice but no impact on colleagues	Replicable across other groups and topic areas

### Knowledge sharing as a mechanism to facilitate change

**Fig. 2:**
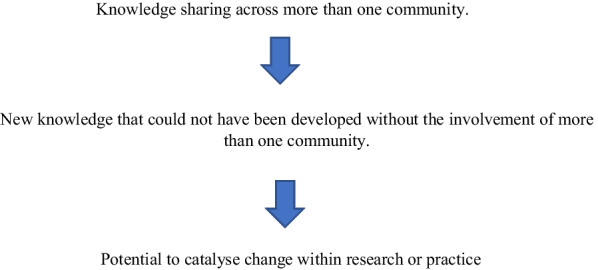
Theory of change model developed to inform initial synthesis

The theory of change identified from a preliminary synthesis of the included studies followed the process outlined within the literature, which is shown in Fig. 2.

All studies confirmed the causal direction of the knowledge sharing mechanism as shown by the arrows in Fig. 2 and were found to be following the process of knowledge sharing across communities with an intention of creating new knowledge (Table 2). Seven studies reported that new knowledge had been created through knowledge sharing [45, 50, 51, 53–55, 57]. However, only three studies attempted to outline the anticipated change from the knowledge-sharing approach [45, 50, 53], and only one study provided any evidence of change [54] (Table 2).

### Evaluation of knowledge sharing technique or approach

Ten studies conducted an evaluation of the knowledge sharing technique or approach to understand its process or effectiveness (perceived or intended) [44–48, 50, 52, 54–56], (Table 4). The other five studies gave detailed descriptive accounts of the knowledge sharing process [20, 49, 51, 53, 57]. There was no relationship between the knowledge-sharing approaches used and whether an evaluation was conducted. Three studies using stakeholder engagement approaches gave a process description [49, 51, 57], one involvement study [53] and one study using an embedded model [20]. Of those studies that conducted an evaluation a range of methodologies were used, which were predominantly qualitative. Six studies used semi-structured interviews [44, 50, 52, 54–56], three studies used mainly observational methods [48, 52, 56], two studies used document analysis [55], two studies used reflective diaries [44, 46] and two studies analysed field notes and emails or meeting recordings [45, 47]. Other methods used were focus groups, surveys and postal questionnaires [45, 48]. Five of the studies that conducted an evaluation of the knowledge sharing technique or approach drew upon a theory or framework to understand or explain the process [46–48, 50, 56] (Table 4).

### Evidence of effectiveness

Of the seven studies that reported the creation of new knowledge [45, 50, 51, 53–55, 57], four also evaluated the process and also attempted to outline the anticipated change from the knowledge-sharing approach [45, 50, 54, 55]. One of these studies used the knowledge-sharing approach of involvement of stakeholders in service design, one explored an organisational collaborative partnership, another used an embedded model and the other a stakeholder engagement approach [45, 50, 54, 55]. The only study that reported a change in practice or research did not outline the process of change and did not explain the process using a theory or framework [54]. However, this study of an embedded model was the only report of a change in practice as a result of a knowledge sharing technique or approach (Table 4).

## Discussion

This review summarises the knowledge sharing techniques and approaches used in NIHR studies between 2006 and 2022. Five knowledge sharing techniques and approaches have been included in NIHR funded health research: embedded models, knowledge brokers, stakeholder engagement, involved research or service design and organisational collaborative partnerships. In applying a mechanism of knowledge sharing, three studies outlined anticipated change from the process of knowledge sharing using the approach of stakeholder involvement [45, 53] and organisational collaborative partnerships [50], and only one study provided evidence of change, which used an embedded model [54].

We found that in some studies knowledge sharing techniques and approaches were used but not identified using established terminology and in other studies terminology was used interchangeably, with a lack of consensus on the definition of terms. This may well reflect the developments overtime in how knowledge is mobilised in a non-linear fashion, as this review included papers from 2008 and tracks the gradual establishment of agreed terminology. However, a current lack of clarity of terms has been identified in the literature around co-design, co-production and co-creation, where terms are used interchangeably and clarity around the aims of the approaches are unclear [72]. This seems also to be the case in what we have referred to as the embedded models, which included researchers in residence and secondment opportunities. It was unclear in synthesising the studies what the different roles were that these terms applied to, as terminology was used differently across the models for example using the term knowledge broker to refer to an embedded researcher working within clinical practice [44].

Knowledge sharing techniques and approaches were often used without reference to underlying theory or an explanation of the anticipated change process. Although an acknowledgment of the clarity provided by a clear theoretical basis to understand the process of knowledge mobilisation has been accepted, this has been relatively recent [73, 74]. Recent studies have highlighted and categorised a large number of theories, models and frameworks available but acknowledged a limited evidence base on their use [75, 76]. In this review, only six studies drew on a theoretical base to explain or predict causality, and only four studies used this for evaluating the knowledge sharing technique or approach. A recent systematic scoping review of knowledge transfer and exchange models also noted a lack of evaluation of the processes and outcomes by those engaged in knowledge mobilisation activities [77]. Evaluation models do exist in the field that construct a framework for assessing impact or change at multiple levels, which also take account of the inherent complexity and uncertainties in assessing change [7]. To encourage greater use of knowledge mobilisation techniques and approaches amongst non-specialists, more explanation of these is needed to facilitate replication with confidence. Studies describing a knowledge sharing technique or approach without reference to an output, outcome or change mechanism, risk losing the interest of the wider research community, as the benefits of this approach are unclear.

This review included studies where knowledge sharing techniques or approaches could be identified but may not necessarily been acknowledged by the authors. Where knowledge-sharing approaches were not acknowledged, the knowledge sharing component was often not reported in detail. For example, in Batchelor 2013, the knowledge-sharing element of the James Lind Alliance Priority Setting Partnership was given little attention in the reporting and was difficult to untangle from the information gathering element of the study [57]. As an older study this may reflect less interest at the time in the process of knowledge sharing with stakeholders, although there were clear attempts to extend the remit of the James Lind Alliance to include researchers in the workshops and to involve stakeholders in designing the research questions. Unfortunately, the lack of detail on the procedure reduces the opportunity for replication or wider evaluation when a project is deemed to be successful, reducing the opportunity for future learning. In work involving public contributors, researchers often gave a more detailed account of process and procedures, which may indicate greater maturity in the field for working with this stakeholder group. This may also give an indication as to why so few studies reported on their knowledge sharing activities and intended impact. As the request from funders for the demonstration of research impact is a relatively new requirement, previous work in this area may not have been seen as important or as a core component of a research study. Likewise, prior to the agreement from funders to fund and support impact related activities such as knowledge mobilisation, achieving impact in services or society may have not been seen as within the remit of the research community to deliver.

Promising techniques and approaches that were evaluated, often focused more on acceptability of the approach rather than whether new knowledge was created. This may have been due to an interest in how to maintain ongoing work with stakeholders, or possibly a lack of confidence in the technique or mechanism leading to new knowledge or in the sensitivity of the evaluation to identify it. Although knowledge sharing can be seen as a simple concept, achieving an authentic approach is known to be a complex process [7, 78]. It is not to suggest that complexity does not exist, only that current reporting may render the purpose of knowledge sharing techniques and approaches invisible to those outside the specialist field. While the importance of identifying and reporting on impact remains a central issue to funders, identifying techniques and approaches that can lead to changes in practice and research will be of value. Currently the NIHR as a funder, requests engagement and impact plans in applications for funding and advocates the use of knowledge mobilisation strategies from the outset of the study to achieve this [18, 79]. Monitoring of the impact from NIHR funded research is then conducted for 5 years after study completion via an online system (Researchfish) [80].

### Strengths and limitations of the review

This systematic review restricted the number of database searches to two and did not explore grey literature, which may have resulted in not identifying all relevant studies. The included studies were also restricted to the English language. However, given that this review is focused on the literature produced by the major UK funder with a requirement for publication in mainstream open access journals, this is less of a concern. A restricted systematic review methodology was used to balance rigour with the resource available [36]. This requires only a proportion of the screening, full-text review and data extraction to be conducted by two reviewers. Given the difficulties with the terminology, unclear methodologies and complex study designs, studies may not have been identified through the initial searches. As outlined earlier, studies often did not report knowledge mobilisation or knowledge sharing activities in a thorough way and this led to difficulties with data extraction and may have led to an underestimation of use of knowledge-sharing approaches. This review specifically focused on the relationship between knowledge sharing as a key element of knowledge mobilisation activity, leading to the creation of new knowledge with the potential to lead to changes in practice or research (impact). Studies that mobilised knowledge for other outcomes were excluded, which may be a weakness in understanding knowledge mobilisation processes more generally. A key strength of this review was the attempt to apply a robust review framework to an often-confusing field of terms and mixed approaches. An established framework was applied to synthesise the current knowledge in this field with the intention to collate the learning to date and to guide those who are not specialists in knowledge mobilisation towards the techniques and approaches which might be useful for future research.

### Key learning

There is a need for clear reporting in the field of knowledge mobilisation that recognises the goals of these techniques and approaches. Theories and models exist that support exploratory work and complex systems, which could be used more widely to explain the knowledge sharing mechanism of knowledge mobilisation approaches. Evaluations of these techniques and approaches could be better linked to the underlying goals or outcomes of change and impact via established theories and explanatory models. This would enable researchers not specialist in the field of knowledge mobilisation to better understand the field and have confidence in introducing these techniques and approaches into their work. Clearer reporting on knowledge sharing processes and outcomes can support the research community and funders alike in identifying where knowledge mobilisation can assist in closing the research to practice gap.

## Conclusions

There is little evidence of the effectiveness of knowledge sharing techniques and approaches used in NIHR research studies in influencing change in practice or ongoing research. This does not mean these techniques and approaches are not effective in instigating change or impacting on practice, rather that clear evidence for this has not yet been produced. Although a complex and often messy field, there are theories, models and frameworks that can be used to shed more light on techniques and approaches that currently show promise but lack evidence for their effectiveness.

### Supplementary Information

**Additional file 1.** Search strategy.

## Data Availability

The datasets used and/or analysed during the current study are available from the corresponding author on reasonable request.

## Abbreviations

NIHR: National Institute for Health and Care Research
CASP: Critical Appraisal Skills Programme
CLAHRC: Collaboration for Leadership in Applied Health Research
