# Supplementary material for: The effectiveness of knowledge-sharing techniques and approaches in research funded by the National Institute for Health and Care Research (NIHR): a systematic review
Source: Health Res Policy Syst. 2024 Apr 2;22:41. doi: 10.1186/s12961-024-01127-5 (PMC10988883; doi:10.1186/s12961-024-01127-5)
Supplement: Supplementary file 1 — Additional file 1. Search strategy. [file 12961_2024_1127_MOESM1_ESM.docx]

Database: Ovid MEDLINE(R) <1946 to present>

Search Strategy:

--------------------------------------------------------------------------------

1 clinical commissioning group.mp.

2 health commissioner.mp.

3 health service manager.mp.

4 policy maker.mp. or Administrative Personnel/

5 clinical commissioning.mp.

6 Policy Making/

7 Health Policy/ or nhs policy.mp.

8 Clinical Governance/

9 Decision Making/

10 "Delivery of Health Care"/

11 policy maker.mp.

12 stakeholder*.mp. or Stakeholder Participation/

13 public contributor.mp.

14 lay person*.mp.

15 Public Opinion/

16 public consultation.mp.

17 Patient Participation/

18 patient involvement.mp.

19 public involvement.mp.

20 Physicians/

21 Consultants/

22 Medical Staff, Hospital/

23 Family Practice/

24 General Practice/ or General Practitioners/

25 clinician.mp. or Nurse Clinicians/

26 hospital manager.mp.

27 service provider*.mp.

28 Nurses/ (37963)

29 1 or 2 or 3 or 4 or 5 or 6 or 7 or 8 or 9 or 10 or 11 or 12 or 13 or 14 or 15 or 16 or 17 or 18 or 19 or 20 or 21 or 22 or 23 or 24 or 25 or 26 or 27 or 28

30 knowledge sharing.mp.

31 Information Dissemination/

32 Cooperative Behavior/

33 "Attitude of Health Personnel"/

34 Interprofessional Relations/

35 knowledge coproduction.mp.

36 knowledge mobili*ation.mp.

37 knowledge transfer.mp.

38 Health Knowledge, Attitudes, Practice/

39 "Diffusion of Innovation"/

40 knowledge exchange.mp.

41 Evidence-Based Medicine/

42 Health Services Research/

43 knowledge translation.mp.

44 knowledge users.mp.

45 Communication/

46 Persuasive Communication/

47 Communication Barriers/

48 Health Communication/

49 personal interaction.mp.

50 30 or 31 or 32 or 33 or 34 or 35 or 36 or 37 or 38 or 39 or 40 or 41 or 42 or 43 or 44 or 45 or 46 or 47 or 48 or 49

51 academic.mp.

52 researcher*.mp.

53 Researcher-Subject Relations/

54 Research Personnel/

55 51 or 52 or 53 or 54

56 29 and 50 and 55

***************************
